# Neurocognitive and mental health outcomes in children with tungiasis: a cross-sectional study in rural Kenya and Uganda

**DOI:** 10.1186/s40249-023-01154-4

**Published:** 2023-11-14

**Authors:** Berrick Otieno, Lynne Elson, Abneel K. Matharu, Naomi Riithi, Esther Chongwo, Khamis Katana, Carophine Nasambu, Francis Mutebi, Herman Feldmeier, Jürgen Krücken, Ulrike Fillinger, Amina Abubakar

**Affiliations:** 1 Kenya Medical Research Institute (KEMRI)-Wellcome Trust Programme, Hospital Road, Kilifi, Kenya; 2 Centre for Tropical Medicine and Global Health, Nuffield Department of Medicine, University of Oxford, Oxford, UK; 3 International Centre of Insect Physiology and Ecology (ICIE), Human Health Theme, Nairobi, Kenya; 4 Institute for Parasitology and Tropical Veterinary Medicine, Freie Universität Berlin, Berlin, Germany; 5 Institute for Human Development, Aga Khan University, Nairobi, Kenya; 6 School of Veterinary Medicine and Animal Resources, College of Veterinary Medicine, Animal Resources and Biosecurity, Makerere University, Kampala , Uganda; 7 Charité University Medicine, Berlin, Germany

**Keywords:** Tungiasis, *Tunga penetrans*, Neglected tropical disease, Neurocognition, Mental health, School-aged children, Africa

## Abstract

**Background:**

Tungiasis, a neglected tropical parasitosis, disproportionately affects children. Few empirical studies have reported neurocognitive and mental health outcomes of children with ectoparasitic skin diseases like tungiasis. Pathophysiology of tungiasis suggests it could detrimentally affect cognition and behaviour. This study pioneered the investigation of neurocognitive and mental health outcomes in children with tungiasis.

**Methods:**

This was a multi-site cross-sectional study including 454 quasi-randomly sampled school-children aged 8–14 from 48 randomly selected schools in two counties in Kenya and a district in Uganda. The participants were stratified into infected and uninfected based on the presence of tungiasis. The infected were further classified into mild and severe infection groups based on the intensity of the infection. Adapted, validated, and standardized measures of cognition and mental health such as Raven Matrices and Child Behaviour Checklist were used to collect data. Statistical tests including a multilevel, generalized mixed-effects linear models with family link set to identity were used to compare the scores of uninfected and infected children and to identify other potential risk factors for neurocognitive and behavioural outcomes.

**Results:**

When adjusted for covariates, mild infection was associated with lower scores in literacy [adjusted *β*(*aβ*) = − 8.9; 95% confidence interval (*CI*) − 17.2, − 0.6], language (*aβ* = − 1.7; *95% CI* − 3.2, − 0.3), cognitive flexibility (*aβ* = − 6.1; *95% CI *− 10.4, − 1.7) and working memory (*aβ* = − 0.3; *95% CI *− 0.6, − 0.1). Severe infection was associated with lower scores in literacy (*aβ* = − 11.0; *95% CI* − 19.3, − 2.8), response inhibition, (*aβ* = − 2.2; *95% CI* − 4.2, − 0.2), fine motor control (*aβ* = − 0.7; *95% CI* − 1.1, − 0.4) and numeracy (*aβ* = − 3; *95% CI* − 5.5, − 0.4).

**Conclusions:**

This study provides first evidence that tungiasis is associated with poor neurocognitive functioning in children. Since tungiasis is a chronic disease with frequent reinfections, such negative effects may potentially impair their development and life achievements.

**Graphical abstract:**

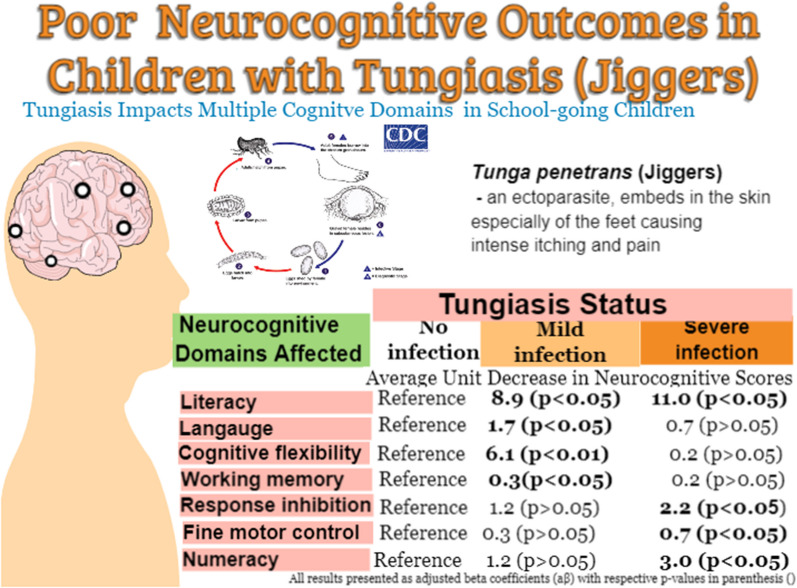

**Supplementary Information:**

The online version contains supplementary material available at 10.1186/s40249-023-01154-4.

## Background

Tungiasis is a common parasitic infection in the tropics. The infection occurs when a female sand flea (*Tunga penetrans*) embeds in the skin. Tungiasis is endemic in South America, the Caribbean, and sub-Saharan Africa and disproportionately affects low-income communities [1]. Particularly children, but also the elderly and individuals with disabilities are the most susceptible to tungiasis [2]. Children are also at critical stage of brain development and are at risk of impaired neurocognitive and mental problems.

Neurocognitive and behavioural development are complex processes that involve distinct yet interrelated bioecological and psychosocial factors. Viral infections like HIV [3] and parasitic infections like schistosomiasis are associated with poor cognitive abilities [4]. Psychosocial factors such as peer, family, and school interactions also exert a significant influence on human cognitive development [5]. Schooling, parenting behaviour, family socioeconomic status, and orphanhood also influence cognitive development in children [6].

Pathogenesis of tungiasis makes it plausible that it can lead to adverse neurocognitive and mental health outcomes. Inflammation at the entry site and bacterial superinfection [7] cause pain and impair mobility and social interactions [8]. Since learning occurs through reciprocal interaction of the individual and the environment [9], impaired interaction puts children with tungiasis at risk for poor neurocognitive development. Although the pathogenesis of tungiasis may contribute to adverse neurocognitive and mental health outcomes among affected persons, there is no implicating empirical evidence. This study investigated the impact of tungiasis on neurocognitive and mental health in school-going children.

## Methods

### Study design and setting

Community-based cross-sectional surveys were implemented as part of a larger study investigating the disease ecology of tungiasis in Matuga and Msambweni sub-counties in Kwale, Kenya; Ugenya sub-county in Siaya; and Bugiri in Uganda. The regions have various cultures and ethnicities, including livestock-keeping practices, soil features, and closeness to animal habitats, yet their climate conditions are comparable. The recruitment of participants and data collection was done between February 2020 and April 2021.

### Study size

The study aimed to test if the mean of outcomes were significantly different in the two group. Therefore, a sample size for a two-sample means test was computed. The study used category fluency as reference outcome. Previous study in a similar setting but with younger population (mean age = 5.2 years) reported a mean category fluency of 15.97 [10]. Assuming a common standard deviation of 2 the study required at least 506 (253 infected and 253 uninfected) participants to detect at least 0.05 difference in means at *α* = 0.05 and power of 0.8. The sample size was calculated using Stata [11]. However, the actual study size was 454 (220 infected and 234 uninfected), mean difference of category fluency between the two group was 2.6 and a common standard deviation of 7, giving the study a power of 0.97.

### Study population and sampling procedure

**Fig. 1:**
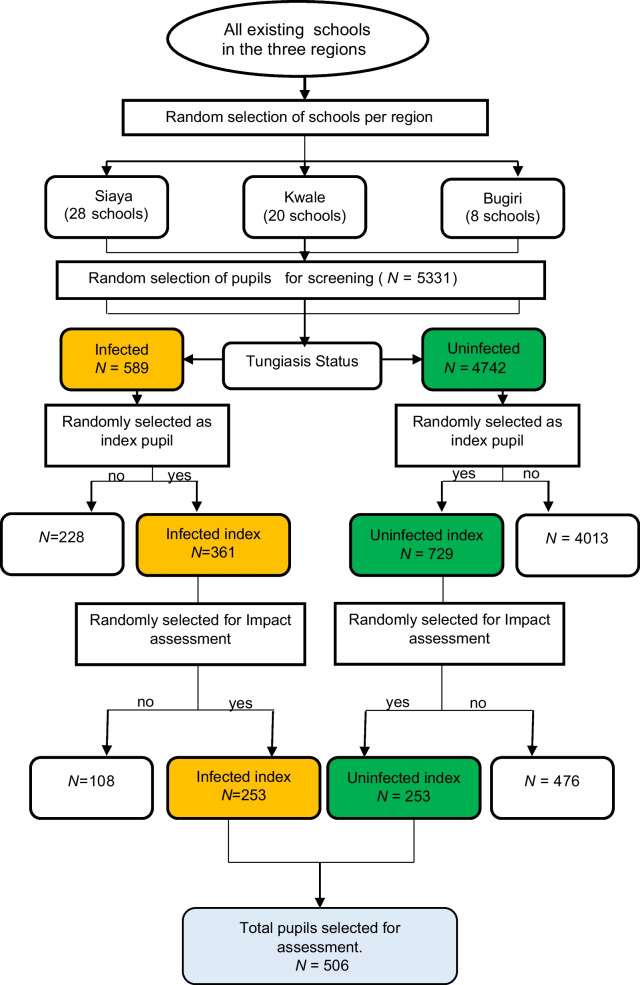
Participant selection flow diagram. Orange represents infected children; green represents uninfected children. Yes, indicates pupils selected for the next stage. No, indicates pupils excluded from the study

The study targeted eight to fourteen-year-old children, the most susceptible to tungiasis infection [2]. The study established inclusion criteria that specified residency in a household with a natural soil floor, given its known association with increased risk of tungiasis, and the availability of an adult caregiver for informed consent and interviews. In addition to those specified for infected participants, eligibility criteria for uninfected participants also required the absence of infected family members. In stage I, sixteen public primary schools were to be randomly chosen within each region from a list of all existing public primary schools. However due to the low prevalence of tungiasis in some regions, additional 12 schools in Siaya and four schools in Kwale were randomly selected. Moreover, due to the exceptionally low prevalence of tungiasis in the Bugiri region, the decision was made to conclude data collection after surveying only eight schools. As a result, the selection outcome for Stage I comprised 28 schools from Siaya, 20 schools from Kwale, and eight schools from Bugiri. In stage II, up to a maximum of 102 school-going children were quasi-randomly selected in each school. This process resulted in a total of 5331 pupils. Hands, and feet of the 5331 pupils were visually inspected for tungiasis. Out of the 5331, 589 pupils were infected while 4742 pupils were uninfected. In each school, up to 10 infected and 20 uninfected pupils were then quasi-randomly selected as index pupils from those with tungiasis and those without, respectively. This process resulted in selection of 361 from 589 infected pupils and 729 pupils out of 4742 uninfected pupils as index pupils as shown in Fig. 1. These index pupils were to participate in the larger study. In stage III, six infected and six uninfected children were quasi-randomly selected in each school from the pool of index pupils. The section of infected pupils at this stage was based on severity of infection-aiming at three with severe (> 10 fleas) and three with a mild infection (< 10 fleas) where possible. Conversely, the selection of uninfected children was done through a simple random process. Overall, stage III resulted in selection of 253 of the 361 infected pupils and 523 of the 729 uninfected pupils. This total of 506 pupils (253 infected and 253 uninfected pupils) formed the the final study group for neurocognitive and mental health assessments (Fig. 1).

### Study variables

Neurocognitive function and mental health problems were main outcome variables. Five domains of neurocognitive functioning were: attention, memory, language, perceptual-motor, and executive function. These domains are detailed in S1 of the Additional file 1. The explanatory variable of interest was tungiasis status. Other explanatory variables included in each model as potential confounders were nutritional status (underweight, stunting, wasting), disability, perinatal complications, residence (Siaya, Kwale, Bugiri), socioeconomic status (SES), school absenteeism (school days missed in the week preceding data collection), orphanhood, household size, ill family member, and both household head-related factors (sex, relation to participant, age) and caregiver-related factors (sex, relation to participant, age, spending time with the participant, exposure to hugging or cuddling, correction method, caregiver depression, and caregiver stress).

### Data sources and methods of measurement

#### Diagnosis of tungiasis and classification of infection

Trained community health workers washed children’s feet to expose embedded fleas. The feet and fingers of the children were visually inspected embedded flea. The selected participants were categorised infected if they had at least an imbedded flea and uninfected if they did not present with a flea. The embedded fleas were manually counted and the infected further categorised into infection status as mild infection if they had less than 10 imbedded fleas and severe infection if they presented with ten or more imbedded fleas.

#### Neurocognitive and mental health measures

Participants underwent approximately two-hour battery of tests administered by trained research assistants to assess their neurocognitive abilities across multiple domains. Language function was evaluated with the Early Grade Reading Assessment (EGRA) [12] and Category Fluency Test (CFT) [13], while attention was assessed with the Comprehensive Trail-Making Test (CTMT) [14] and Stroop Color and Word Test (SCWT) [15]. Working memory was evaluated using the backward digit span task [16], and fine motor control was assessed with the bead threading test [17]. The battery also included the Early Grade Maths Assessments (EGMA) [18], Standard and Coloured Raven Progressive Matrices (RPM) [19] to evaluate numeracy and nonverbal intelligence, respectively. The lower scores in these cognitive tests indicate poor neurocognitive function. These tests are valid and reliable for assessing their respective domains and have been adapted for use in Kenya [20] and Uganda [10]. The Child Behavior Checklist [CBCL] [21] was used to assess mental health outcomes. In this study, the total score was used to assess mental health problems, with higher scores indicating more problems. The neurocognitive and mental health measures are detailed in S1 in supplementary material.

#### Covariates

Anthropometric measurements including height, weight, and Mid-arm circumference (MUAC) were used to assess nutritional status. Height was measured using a stadiometer, weight was measured using a calibrated scale, and MUAC was measured using a flexible tape measure. Height-for-age (HAZ) and weight-for-age (WAZ) *z*-scores were calculated according to the Centers for Disease Control and Prevention (CDC) [22], with HAZ *z-*scores < − 2 and WAZ *z-*scores < − 2 indicating stunting and underweight, respectively, while MUAC was used to evaluate wasting. Structured questionnaires were used to collect data on factors associated with poor neurocognitive and mental health outcomes, including disability child perinatal complications, region of residence, child age, child sex, and school grade level. Household socioeconomic status (SES) was assessed using tetrachoric principal component analysis (PCA) and the resulting wealth index was created based on eigenvalue and scree plot as detailed in S2, S3 and S4 in the Additional file 1. Psychosocial covariates including orphanhood, school absenteeism, household size, and caregiver information were also collected. Respondents’ relation to participant, age, spending time with the participant, exposure to hugging or cuddling, correction method, and caregiver mental health (depression, and stress).

Psychosocial covariates covered various topics such as orphanhood (also assessed using binary response options), school absenteeism (measured as the number of days absent from school in the week preceding data collection), household size (classified as either more than 2 adults or less than 2 adults), having a chronically ill family member (also assessed using binary response options), sex of household head, relation of the household head to the child (classified as either "child" or "not child"), age of household head, sex of caregiver, age of caregiver, and the relation of the caregiver to the child. Other factors assessed in the questionnaires included the amount of time the caregiver spent with the child (reported as "a lot of time" or "not a lot of time"), whether the child was hugged or cuddled (assessed using a binary response option of "yes" or "no"), and the caregiver's methods of correcting the child (reported as "beating" or "other methods").

Caregiver mental health was measured using Patient Health Questionnaire-9 (PHQ-9) and Parental Stress Scale (PSS). The PHQ-9 is a self-report questionnaire that measures depression by asking the respondent to rate the frequency of their symptoms over the past 2 weeks on a scale of 0–3 [23]. The questionnaire covers various areas related to depression, and scores range from 0 to 27. In this study depression cutoff was scores of 10 and above. The Parental Stress Scale (PSS), a questionnaire used to assess parental stress, includes 18 items covering various aspects of parenting, and respondents rate how often they experience stress related to each item on a 5-point scale [24]. The total score is calculated by summing the scores, with higher scores indicating higher levels of parental stress. In this study CBCL, PHQ-9 and PSS had acceptable alphas of 0.94, 0.87 and 0.68, respectively an indication of internal consistency.

### Measures to address bias and errors

The outcome assessors were distinct from the infection assessors and were kept unaware of the participant's status to minimize potential biases, however, the status could be known in participants with visible signs of infection. Questionnaires and test score sheets were adapted to the Research Electronic Data Capture (REDCap) database [25], hosted at the International Centre of Insect Physiology and Ecology (ICIPE). Responses were recorded on tablets conditionally formatted to ensure the validity, accuracy, and completeness of the data. The study's data collection involved in-person interviews and assessments performed by assessors trained in neurocognitive evaluations. To accommodate diverse local languages, the questionnaires were initially written in English and subsequently translated into Kiswahili, Dholuo, and Kisoga. Information bias was reduced through pretesting, adjustments, and clarifications of the questions before commencing data collection. To promote consistency in the interview process, a workshop was held involving all assessors and investigators to establish a common interpretation of the responses. Moreover, to minimize misclassification bias, the assessors verified the presence of other infection indicators (like pain and itching) in infected.

### Statistical analysis

Data were analysed utilizing STATA software version 15 [11]. The difference in distributions between the uninfected group and each infected group, as well as among all groups together, were compared using binomial and multinomial tests, respectively. Continuous data were presented as means and standard errors (*SE*) if normally distributed or medians and interquartile range (IQR) if skewed. For bivariate analyses of continuous data, Mann Whitney U Test, or the Kruskal–Wallis rank test was used to compare skewed distributions while Two Sample Students *t* test or analysis of variance (ANOVA) was used to compare means of normally distributed data. Categorical data were presented as frequencies with respective percentages and their proportions compared using the Pearson Chi-Square Test or Fisher's Exact Test. Analysis of covariates (ANCOVA), followed by the Scheffe Test was used for pairwise comparison of adjusted means. Covariates adjusted for included age, sex, grade, nutritional status, care giver education, disability, school absenteeism and SES.

For regression analyses, multilevel mixed effects generalized linear models with gaussian family and identity link were used for bivariable and multivariable analyses with the unique school identifier as a random effect to identify factors associated with neurocognitive and mental health outcome scores in children adjusting for age, sex, and grade as priori confounders. Multivariate analyses were conducted separately for each neurocognitive and mental health outcome. Backward stepwise selection was used to identify the most significant variables for the model. An exhaustive model containing all predictor variables was initially established. The variable with the highest p-value was subsequently eliminated from the model. This iterative process was continued until the stopping criterion (*P* < 0.05) was reached and the model selected as final.

## Results

### Characteristics of participants

This study involved 506 participants, however 52 (approximately 10%) had crucial variables like age, sex or grade missing at random and were excluded from analysis. The exclusion resulted in imbalanced infected and uninfected groups. Analysis was done on data from 454 participants, including 234 uninfected children, 109 children with mild infection, and 111 with severe infection (Table 1).

**Table 1:** Socio-demographic, biological, and psychosocial characteristics of participants—comparison between uninfected, mild disease, and severe disease groups

Characteristics of participants (all variables presented as *N* (%) unless specified)			Total *N* = 454	Uninfected *N* = 234	Mild disease *N* = 109	Severe disease *N* = 111	Uninfected vs Mild disease *P-Value*	Uninfected vs severe disease *P-Value*	Overall Comparison statistic *P-Value*
Socio-demographic characteristics									
Residence	Siaya		208 (45.8)	105 (44.8)	45 (41.3)	58 (52.3)	0.501	0.001^a^	< 0.001^a^
	Kwale		199 (43.8)	101(43.2)	46 (42.2)	52 (46.8)			
	Bugiri		47 (10.4)	28 (12.0)	18 (16.5)	1 (0.9)			
Child’s age in years, median (IQR)			11.0 (9.0*–*12.0)	11.0 (10.0*–*13.0)	10.0 (9.0*–*12.0)	10.0 (9.0*–*12.0)	< 0.001^b^	0.011^b^	< 0.001^c^
Child’s sex	Females		185 (42.7)	128 (54.7)	33(30.3)	30 (27.0)	< 0.001	< 0.001	< 0.001
	Males		248 (57.3)	106 (45.3)	76 (69.7)	81 (73.0)			
School grade	Lower (Grade 1 to grade 4)		316 (73.0)	146 (62.4)	92 (84.4)	94 (84.7)	< 0.001	< 0.001	< 0.001
	Upper (Grade 5 to class 8)		117 (27.0)	88 (37.6)	17 (15.6)	17 (15.3)			
SES, mean (SD)			1.8 (0.7)	1.8 (0.7)	1.8 (0.7)	1.8 (0.7)	0.837^d^	0.850^d^	0.4787^e^
Biological characteristics									
Nutritional status	Underweight	No	394 (86.8)	211 (90.2)	93 (85.3)	90 (81.1)	0.188	0.018	0.058
		Yes	60 (13.2)	23 (9.8)	16 (14.7)	21 (18.9)			
	Stunting	No	403(88.8)	211 (90.2)	94 (86.2)	98 (88.3)	0.280	0.593	0.552
		Yes	51 (11.2)	23 (9.8)	15 (13.8)	13 (11.7)			
	MUAC		18.9 (17.5*–*20)	19.0(18.0*–*20.5)	18.0 (17.0*–*19.9)	18.0 (17.0*–*19.3)	< 0.001^b^	< 0.001^b^	< 0.001^c^
Disability	No		431 (94.9)	224 (95.7)	101 (92.7)	106 (95.5)	0.236	0.461	0.461
	Yes		23 (5.1)	10 (4.3)	8 (7.3)	5 (4.5)			
Perinatal complications	No		400 (88.1)	208 (88.9)	98(89.9)	94 (84.7)	0.777	0.269	0.424
	Yes		54 (11.9)	26 (11.1)	11 (10.1)	17 (15.3)			
Psychosocial characteristics									
Absenteeism	No		438 (96.5)	223(95.3)	106 (97.2)	109 (98.2)	0.561^a^	0.237^a^	0.396^a^
	Yes		16 (3.5)	11 (4.7)	3 (2.8)	2 (1.8)			
Orphan hood	No		424 (93.4)	222 (94.9)	102 (93.6)	100 (90.1)	0.626	0.096	0.247
	Yes		30 (6.6)	12 (5.1)	7 (6.4)	11 (9.9)			
Household size	Having two or less adults		288 (63.4)	144 (61.5)	71 (65.1)	63 (56.8)	0.521	0.397	0.439
	Having more than two adults		166 (36.6)	90 (38.5)	38 (34.9)	48 (43.2)			
Family ill member	No		282 (63.0)	152 (65.0)	73 (67.0)	63 (56.8)	0.715	0.142	0.228
	Yes		166 (37.0)	82 (35.0)	36 (33.0)	48 (43.2)			
HH head factors	Sex of HH	Female	93 (20.5)	49 (20.9)	22 (20.2)	22 (19.8)	0.872	0.810	0.968
		Male	361 (79.5)	185 (79.1)	87 (79.8)	89 (80.2)			
	Relation to child	Not biological	122 (26.9)	62 (26.5)	28 (25.7)	32 (28.8)	0.874	0.649	0.856
		Biological	332 (73.1)	172 (73.5)	81 (74.3)	79 (71.2)			
	Age of HH, median (IQR)		46 (40*–*57)	45 (40*–*56)	45 (40*–*57)	48 (40*–*62)	0.841^b^	0.185^b^	0.4156^c^
Caregiver factors	Sex of CG	Female	417 (91.9)	222 (94.9)	97 (89.0)	98 (88.3)	0.872	0.028	0.052
		Male	37 (8.2)	12 (5.1)	12 (11.0)	13 (11.8)			
	Relation to child	Other	168 (37.0)	88 (37.6)	43 (39.4)	37 (33.3)	0.744	0.440	0.619
		Mother	286 (63.0)	146 (62.4)	66 (60.6)	74 (66.7)			
	Age of CG, median (IQR)		37 (30*–*45)	37 (30*–*45)	38 (30*–*45)	36 (30*–*45)	0.710^b^	0.645^b^	0.8730^c^
	Time with child	Not a lot	299 (67.8)	153 (65.4)	75 (68.8)	84 (75.7)	0.532	0.054	0.156
		A lot	142 (32.2)	81 (34.6)	34 (31.2)	27 (24.3)			
	Cuddle or Hug	No	318 (70.0)	166 (70.1)	71 (65.1)	81 (73.0)	0.279	0.696	0.408
		Yes	136 (30.0)	68 (29.1)	38 (34.9)	30 (27.0)			
	Child correction	Beating	298 (65.6)	152 (65.0)	76 (69.7)	70 (63.1)	0.384	0.731	0.554
		Others	156 (34.4)	82 (35.0)	33 (30.3)	41 (36.9)			
	CG Depression	No	269 (59.3)	151 (64.5)	60 (55.1)	58 (52.3)	0.093	0.029	0.056
		Yes	185 (40.7)	83 (35.5)	49 (44.9)	53 (47.7)			
	CG PSS scores, median (IQR)		47 (42*–*51)	46 (41*–*50)	48 (42*–*51)	48 (42*–*51)	0.170^b^	0.131^b^	0.208^c^

*SES* Socioeconomic status, *SD* Standard deviation, *IQR* Interquartile range, *MUAC* Mid-Upper Arm Circumference, *HH* Household head, *CG* Caregiver, *PSS* Parent stress scale, explain the p-values

Significant p-values and in bold. All *P*-values from Chi square unless specified

^a^Fisher’s exact test

^b^Mann Whitney U Test

^c^Kruskal Wallis test

^d^Two sample Student’s t test

^e^ANOVA

The three groups; uninfected, mild infection and severe infection were differently disturbed by sex, age, grade and MUAC (*P* < 0.001) as shown in Table 1. A higher proportion of participants with severe disease were underweight than the uninfected (18*.*9% vs 9.8%; *P* = 0*.*018). Similarly, higher proportion severe infection group had male caregivers [13/111(11*.*8%)] than infected [12/109 (5*.*1%)]. Compared to the uninfected, the severe infection group had a higher proportion of caregivers with depression (47*.*3% vs 35*.*5%; *P* = 0*.*029). Other characteristics of participants are presented in Table 1.

### Neurocognitive and behavioural outcomes

In the current study, the infected group had significantly lower scores than the uninfected group in literacy, language, response inhibition, working memory, fine motor control, non-verbal intelligence, and numeracy, as detailed in Table 2. The lower neurocognitive tests scores suggest impaired neurocognitive ability. Infected group had higher mean total problem scores than uninfected group (34*.*4 vs 32*.*2) indicating the presence of behavioural and emotional issues. The uninfected, mild infection and severe infections groups had significantly different adjusted means in literacy, language, cognitive flexibility, working memory, fine motor, and behavioral problems after adjusting for included age, sex, grade, nutritional status, care giver education, disability, school absenteeism and SES. Compared to uninfected, mild infection group had significantly lower scores in literacy, language, cognitive flexibility, working memory, and fine motor while severe infection had significantly lower scores in literacy, fine motor, and behavioral problems as shown in Table 3.

**Table 2:** Comparison of neurocognitive and behavioral outcomes between uninfected and infected groups—summary of main domains and sub-domains scores

Outcomes		Overall, *N* = 454			Uninfected, *N* = 234 Mean (*SE*)	Infected, *N* = 220 Mean (*SE*)	Difference in means (95% *CI*)^b^	*P*
Main domains	Assessed sub-domains	Min	Max	Mean (se)				
Language	Literacy ability	8	142	94.6 (2.0)	103.6 (2.6)	85.0 (3.1)	18.6 (10.7–26.4)	< 0.001^a^
	Language	6	58	23.8 (0.3)	25.0 (0.5)	22.4 (0.5)	2.6 (1.3–3.9)	< 0.001
Attention	Cognitive flexibility	6	140.7	40.5 (1)	42.0 (1.3)	38.9 (1.5)	3.1 (− 0.8 to 7.0)	0.118^a^
	Response inhibition	20	80	37.1 (0.4)	38.2 (0.6)	36 (0.6)	2.2 (0.5–3.9)	0.011
Memory	Working memory	0	9	2.8 (0.1)	3 (0.1)	2.5 (0.1)	0.5 (0.2–0.7)	< 0.001
Perceptual*-*motor	Fine motor ability	4	15.3	9.9 (0.1)	10.3 (0.1)	9.4 (0.1)	0.9 (0.5–1.3)	< 0.001
Executive functioning	Nonverbal intelligence	0	43	13.4 (0.4)	14.3 (0.5)	12.3 (0.4)	2 (0.6–3.4)	0.004^a^
	Numeracy ability	0	50	39.6 (0.6)	41.4 (0.7)	37.7 (0.9)	3.7 (1.4–6.0)	0.002^a^
Behavioral outcome								
Total behavioral problems		0	126	32.2(1)	30.1 (1.3)	34.4 (1.6)	− 4.3 (− 8.4, − 0.1)	0.043^a^

All p-values are from two sample Student’s t test unless specified

^a^Welch’s t-test

^b^Difference (score uninfected – score uninfected)

**Table 3:** Comparison of neurocognitive and behavioral outcomes among uninfected, mild tungiasis, and severe tungiasis groups—adjusted means and group differences corrected for effects of covariates

Outcomes		Uninfected	Mild tungiasis	Severe tungiasis	Overall group differences				Uninfected vs mild disease P-value*	Uninfected vs severe disease P-value*
Main domain	Sub-domain	Adjusted mean (*SE*)	Adjusted mean (*SE*)	Adjusted mean (*SE*)	F	df	*P*-value	Partial eta squared		
Language	Literacy ability	99.0 (2.61)	86.1 (3.78)	93.8 (3.75)	3.73	2,434	0.024	0.016	< 0.001	0.014
	Language	24.4 (0.44)	22.1 (0.63)	24.2 (0.63)	4.53	2,434	0.011	0.02	< 0.001	0.169
Attention	Cognitive flexibility	41.3 (1.35)	35.2 (1.96)	43.8 (1.95)	5.35	2,434	< 0.001	0.024	0.008	0.873
	Response inhibition	37.7 (0.59)	36.6 (0.86)	36.4 (0.85)	1.03	2,434	0.356	0.004		
Memory	Working memory	2.9 (0.09)	2.5 (0.12)	2.7 (0.12)	3.61	2,434	0.027	0.016	0.001	0.097
Perceptual-motor	Fine motor ability	10.1 (0.11)	9.8 (0.16)	9.5 (0.16)	4.37	2,434	0.013	0.02	0.002	< 0.001
Executive functioning	Nonverbal intelligence	13.2 (0.37)	13.3 (0.54)	13.8 (0.53)	0.34	2,434	0.710	0.001		
	Numeracy ability	40.4 (0.77)	39.1 (1.12)	38.5 (1.11)	1.07	*2,434*	0.342	0.004		
Behavioral outcomes										
Total behavioral problems		30.0 (1.49)	31.8 (2.16)	37.1 (2.14)	3.51	2,434	0.03	0.016	0.858	0.023

All *P*-values are from Analysis of Covariance. Covariates adjusted for included age, sex, grade, nutritional status, care giver education, disability, school absenteeism and socioeconomic status

*SE* standard error

*Scheffe Test for pairwise comparison of means

### Covariates of neurocognitive and mental health outcomes

In the bivariable regression analyses, mild infection was associated with lower scores in literacy, language, cognitive flexibility, and working memory, while severe disease was associated with lower scores in fine motor control and higher scores in behavioural problems (Table 4) Other factors associated with lower neurocognitive and behavioural problem are shown in Table 4. After controlling for covariates in multivariable analyses, the mild infection was associated with significant lower scores in literacy [adjusted beta co-efficient (*aβ*) = − 8*.*9; 95% confidence interval (*CI*): − 17*.*2, *− *0*.*6] where on average, there was 8*.*9 unit decrease in literacy score in mild infection compared to uninfected as shown in Table 5. Similarly, mild infection was associated with 1*.*7 (*aβ* = − 1*.*7; *95% CI *− 3*.*2, *− *0*.*3), 6*.*1 (*aβ* = − 6*.*1; *95% CI *− 10*.*4, − 1*.*7) and 0*.*3 (*aβ* = − 0*.*3; *95% CI −* 0*.*6, *− *0*.*1) unit decrease in language cognitive flexibility and working memory, respectively (Table 5). Severe infection was associated with significant lower scores in response inhibition, fine motor control, and numeracy. Averagely, severe infection was associated with a 2.2 unit decrease in response inhibition (*aβ* = − 2*.*2; *95% CI *− 4*.*2, *− *0*.*2) a 0.7 unit decrease in fine motor control (*aβ* = − 0*.*7; *95% CI* − 1*.*1, − 0*.*4) and a 3 unit decrease in numeracy (*aβ* = − 3; *95% CI* − 5*.*5, − 0*.*4) (Table 5). Stunting was associated with lower scores in language (*aβ* = − 2*.*0; *95% CI* − 3*.*9, − 0*.*2) and numeracy (*aβ* = − 5*.*7; *95% CI* − 9, − 2*.*4). Unexpectedly wasting and perinatal complications were associated with better response inhibition scores. Other factors independently associated with lower scores in various domains included residing in a specific geographic region, belonging to households of larger size, having a chronically ill family member, relation to the household head, caregiver depression and caregiver stress (Table 5). After adjusting for covariates, mental health outcomes were neither associated with mild nor severe infection. However, residing in Kwale (*aβ* = 9*.*1; *95% CI* 3*.*5, 14*.*7), having an ill family member (*aβ* = 4*.*5; *95% CI* 0*.*7, 8*.*2), caregiver depression (*aβ* = 11*.*4; *95% CI* 7*.*7, 15*.*2), and caregiver stress (*aβ* = 0*.*4; *95% CI* 0*.*1, 0*.*6) remained significantly associated with mental health outcomes.

**Table 4:** Beta coefficients (*β*) and 95% confidence interval (*CIs*) for the association of characteristics of participants with neurocognitive and behavioral outcomes (*N* = 454)

Risk factors		Language		Attention		Memory	Perceptual-motor	Executive functioning	Behavioral problems		
		Literacy	Language	Cognitive flexibility	Response inhibition	Working memory	Fine motor	Non-verbal intelligence	Numeracy		Total problems
Biological factors											
Tungiasis infection status	Control	Reference	Reference	Reference	Reference	Reference	Reference	Reference	Reference		Reference
	Mild	− 11.7 (− 20.4*, − *3)**	− 2.2 (− 3.7*, − *0.6)**	− 6.7 (− 11.3*, − *2)**	− 1.6 (− 3.8 to 0.5)	− 0.4 (− 0.7*, − *0.1)*	− 0.3 (− 0.7 to 0.1)	0.3 (− 1 to 1.6)	− 1.2 (− 3.9 to 1.4)		1.9 (− 2.7 to 6.5)
	Severe	− 6.6 (− 15.3 to 2.1)	− 0.2 (− 1.7 to 1.4)	2.1 (− 2.5 to 6.8)	− 1.4 (− 3.5 to 0.7)	− 0.1 (− 0.4 to 0.1)	− 0.6 (− 1*, − *0.2)**	0.9 (− 0.4 to 2.2)	− 1.8 (− 4.5 to 0.8)		4.7 (0 to 9.3)*
Nutritional status	Underweight	− 5.6 (− 16.2 to 5.0)	− 1.2 (− 3 to 0.7)	− 2.1 (− 7.8 to 3.5)	− 2.4 (− 4.9 to 0.1)	− 0.2 (− 0.5 to 0.2)	− 0.3 (− 0.7 to 0.2)	0.9 (− 0.6 to 2.5)	− 3.2 (− 6.4*, − *0.1)*		0.6 (− 5.1 to 6.2)
	Stunting	− 12.3 (− 23.5*, − *1.1)*	− 2.5 (− 4.5*, − *0.6)*	0.3 (− 5.7 to 6.4)	− 0.8 (− 3.5 to 1.9)	− 0.4 (− 0.8*, − *0.1)*	− 0.5 (− 1 to 0)	− 0.6 (− 2.2 to 1.1)	− 5.4 (− 8.8*, − *2.1)**		0.5 (− 5.5 to 6.5)
	Wasting	0.2 (− 1.9 to 2.2)	0.3 (0 to 0.7)	0.3 (− 0.8 to 1.4)	0.6 (0.2 to 1.1)**	0 (0 to 0.1)	0 (− 0.1 to 0.1)	− 0.1 (− 0.4 to 0.2)	0.1 (− 0.5 to 0.7)		− 0.5 (− 1.6 to 0.6)
Disability (yes)		−4.8(−20.5 to 11.0)	− 2.2 (− 4.9 to 0.6)	− 6.9 (− 15.3 to 1.6)	− 0.9 (− 4.7 to 2.9)	− 0.3 (− 0.9 to 0.2)	− 0.5 (− 1.2 to 0.2)	− 1.6 (− 3.9 to 0.7)	− 3.3 (− 8 to 1.5)		0.1 (− 8.3 to 8.4)
Perinatal complications (Yes)		7.2(− 3.5 to 17.9)	1.4 (− 0.5 to 3.2)	2.1 (− 3.6 to 7.9)	3.7 (1.2 to 6.2)**	0.1 (− 0.2 to 0.5)	− 0.1 (− 0.6 to 0.4)	1.7 (0.1 to 3.2)	0.6 (− 2.7 to 3.8)		0.5 (− 5.2 to 6.2)
Sociodemographic factors											
Residence	Siaya	Reference	Reference	Reference	Reference	Reference	Reference	Reference	Reference		Reference
	Kwale	2.3 (− 7.4 to 11.9)	− 2.3 (− 3.6*, − *0.9)**	− 11 (− 16.1*, − *6)**	− 1.5 (− 3.3 to 0.3)	− 0.2 (− 0.5 to 0.1)	0.1 (− 0.2 to 0.4)	1.3 (0.1 to 2.5)*	− 0.5 (− 3.3 to 2.4)		11.8 (5.3 to 18.3)**
	Bugiri	− 44.6 (− 57.2*, − *32.1)**	− 6.7 (− 8.8*, − *4.6)**	− 15.3 (− 22.3*, − *8.2)**	− 6.7 (− 9.6*, − *3.8)**	− 0.9 (− 1.3*, − *0.5)**	− 1 (− 1.5*, − *0.5)**	− 1.5 (− 3.3 to 0.4)	− 6 (− 9.9*, − *2.1)**		− 8.4 (− 15.6*, − *1.1)
SES		3.2(− 2.3 to 8.8)	1.3 (0.4 to 2.2)**	5.4 (2.5 to 8.3)**	0.6 (− 0.6 to 1.8)	0.2 (0 to 0.4)*	0.1 (− 0.1 to 0.3)	0.2 (− 0.6 to 1.0)	0.8 (− 0.8 to 2.4)		− 2.5 (− 5.6 to 0.7)
Psychosocial factors											
School absenteeism (Yes)		− 23.4 (− 42.4*, − *4.3)*	− 3.8 (− 7.1*, − *0.5)*	− 0.4 (− 10.7 to 9.9)	− 3.3 (− 7.8 to 1.2)	− 0.7 (− 1.3*, − *0)*	− 0.2 (− 1.0 to 0.7)	1.8 (− 1.0 to 4.6)	2 (1.3 to 2.7)		1.7 (− 8.5 to 11.9)
Orphan hood (Yes)		− 11.7 (− 25.9 to 2.4)	0 (− 2.5 to 2.5)	2.4 (− 5.2 to 10.1)	− 1 (− 4.4 to 2.4)	− 0.3 (− 0.7 to 0.2)	− 0.1 (− 0.8 to 0.5)	− 1 (− 3.1 to 1.1)	− 1.9 (− 7.7 to 3.9)		6.3 (− 1.3 to 13.9)
Household size (> 2 adults)		− 6.2 (− 13.4 to 0.9)	− 0.7 (− 2 to 0.5)	− 1.3 (− 5.1 to 2.6)	− 1.1 (− 2.8 to 0.6)	− 0.3 (− 0.5*, − *0)*	0 (− 0.3 to 0.4)	0.8 (− 0.2 to 1.9)	− 4.5 (− 8.8*, − *0.3)*		0.1 (− 3.7 to 3.9)
Ill family member (Yes)		− 3.7 (− 17.3 to 3.7)	1 (− 0.3 to 2.4)	9.2 (5.3 to 13.2)**	− 2.1 (− 3.8*, − *0.4)*	− 0.1 (− 0.3 to 0.2)	− 0.2 (− 0.6 to 0.1)	0.1 (− 1.0 to 1.2)	− 1.6 (− 3.8 to 0.6)		5.2 (1.1 to 9.2)*
HH factors	Sex of HH head (male)	4 (− 4.5 to 12.5)	− 1.3 (− 2.8 to 0.2)	1.7 (− 2.9 to 6.3)	− 1.1 (− 3.1 to 1.0)	0.2 (− 0.1 to 0.4)	0.2 (− 0.1 to 0.6)	− 0.5 (− 1.8 to 0.7)	− 1.2 (− 3.4 to 1.1)		− 1.6 (− 6.1 to 3.0)
	Relation of HH head to child (child)	− 5.3 (− 13.0 to 2.5)	− 0.3 (− 1.7 to 1.1)	0.6 (− 3.6 to 4.8)	− 0.8 (− 2.7 to 1.0)	0.1 (− 0.1 to 0.4)	− 0.1 (− 0.4 to 0.3)	− 0.3 (− 1.4 to 0.9)	1.1 (− 1.5 to 3.7)		2.6 (− 1.5 to 6.7)
	Age of HH head	0.0 (− 0.2 to 0.3)	0 (0 to 0.1)	0 (− 0.1 to 0.2)	0 (0 to 0.1)	0 (0 to 0)	0 (0 to 0)	0 (0 to 0.1)	− 0.8 (− 3.2 to 1.5)		− 0.1 (− 0.2 to 0)
CG factors	Sex of CG (male)	7.3 (− 5.3 to 20.1)	− 0.9 (− 3.1 to 1.3)	0.6 (− 6.3 to 7.4)	1.9 (− 1.2 to 4.9)	0.5 (0.1 to 0.9)*	− 0.1 (− 0.7 to 0.4)	− 1 (− 2.8 to 0.9)	0 (− 0.1 to 0.1)		− 0.9 (− 7.7 to 5.9)
	Relation of CG to child (mother)	0.5 (− 7.7 to 6.8)	1.3 (0.1 to 2.6)*	2.4 (− 1.5 to 6.2)	0.9 (− 0.8 to 2.6)	0.1 (− 0.1 to 0.4)	0.1 (− 0.2 to 0.4)	0 (− 1.1 to 1.1)	1.6 (− 2.3 to 5.4)		− 0.5 (− 4.4 to 3.3)
	Age of CG	− 0.1 (− 0.2 to 0.3)	0 (0 to 0.1)	0 (− 0.1 to 0.2)	0 (0 to 0.1)	0 (0 to 0)	0 (0 to 0)	0 (0 to 0.1)	− 0.2 (− 2.4 to 2.0)		− 0.2 (− 0.3 to 0)*
	Time CG with child (a lot)	− 2.9 (− 10.5 to 4.8)	− 0.3 (− 1.6 to 1)	− 3.9 (− 7.9 to 0.2)	− 0.4 (− 2.2 to 1.4)	0.2 (− 0.1 to 0.4)	− 0.1 (− 0.4 to 0.3)	− 0.1 (− 1.2 to 1.1)	0 (− 0.1 to 0.1)		− 5.1 (− 9.1*, − *1)*
	CG Cuddle/Hug (Yes)	− 7.2 (− 15.2 to 0.9)	− 1.6 (− 3*, − *0.2)*	− 4.9 (− 9.1*, − *0.6)*	− 0.6 (− 2.5 to 1.3)	0.2 (− 0.1 to 0.4)	− 0.1 (− 0.5 to 0.2)	− 0.8 (− 2 to 0.4)	0.8 (− 1.5 to 3.1)		− 0.8 (− 5.3 to 3.6)
	CG Child correction (others)	5.8 (− 1.6 to 12.3)	− 0.5 (− 1.8 to 0.8)	− 1.1 (− 5.1 to 2.9)	2.4 (0.6 to 4.1)	0 (− 0.3 to 0.2)	0.1 (− 0.2 to 0.4)	− 0.1 (− 1.2 to 1.0)	− 1.1 (− 3.5 to 1.4)		− 0.3 (− 4.3 to 3.7)
	CG depression	− 5.4 (− 12.7 to 1.9)	− 0.9 (− 2.1 to 0.4)	− 2 (− 5.9 to 1.9)	− 1.3 (− 3.0 to 0.4)	− 0.3 (− 0.5*, − *0.1)*	− 0.6 (− 0.9*, − *0.3)**	0.3 (− 0.7 to 1.4)	1.1 (− 1.2 to 3.4)		11.3 (7.6 to 15.1)**
	CG stress	− 0.2 (− 0.6 to 0.2)	− 0.1 (− 0.2 to 0)*	0 (− 0.2 to 0.3)	− 0.2 (− 0.3*, − *0.1)**	0 (0 to 0)**	0 (0 to 0)	0 (0 to 0.1)	− 1.1 (− 3.3 to 1.1)		0.4 (0.1 to 0.6)**

All results presented as β(95%CI)

*SES* socioeconomic status, *HH* household head, *CG* caregiver, *AIC* Akaike's information criteria

*P < 0.05,**P < 0.01

**Table 5:** Adjusted beta coefficients (*aβ*) and 95% confidence intervals (*CIs*) for the association of characteristics of participants with neurocognitive and behavioral outcomes (*n* = 454)

Risk factors		Language		Attention		Memory		Perceptual-motor	Executive functioning	Abnormal behavior
		Literacy	Language	Cognitive flexibility	Response inhibition	Working memory	Fine motor control	Non-verbal intelligence	Numeracy	Total problems
Biological factors										
Tungiasis infection status	Control	Reference	Reference	Reference	Reference	Reference	Reference	Reference	Reference	Reference
	Mild	− 8.9 (− 17.2, − 0.6)*	− 1.7 (− 3.2, − 0.3)*	− 6.1 (− 10.4, − 1.7)**	− 1.2 (− 3.1–0.8)	− 0.3 (0.6, − 0.1)*	− 0.3 (0.6*–*0.1)	0.2 (− 1.1*–*1.4)	− 1.2 (− 3.7*–*1.4)	1.1 (− 3*–*5.3)
	Severe	− 11.0 (− 19.3*, − *2.8)**	− 0.7 (− 2.1*–*0.8)	0.2 (− 4.5*–*4.2)	− 2.2 (− 4.2*, − *0.2) *	− 0.2 (− 0.5*–*0.1)	− 0.7 (− 1.1*, − *0.4)**	0.7 (− 0.5*–*2.0)	− 3 (− 5.5*, − *0.4)*	1.7 (− 2.5*–*5.9)
Nutritional status	Stunting	.	− 2.0 (− 3.9*, − *0.2)*						− 5.7 (− 9*, − *2.4)**	
	Wasting				0.6 (0.1*–*1)**					
Perinatal complications (yes)					3.5 (1.1*–*5.9)**					.
Sociodemographic factors										
Residence	Siaya	Reference	Reference	Reference	Reference	Reference	Reference	Reference	Reference	Reference
	Kwale	.	− 2.1 (− 3.4*, − *0.8)**	− 10.1 (− 15.6*, − *4.5)**				1.6 (0.4*–*2.8)*	.	9.1 (3.5*–*14.7)**
	Bugiri	− 47.5 (− 59.8*, − *35.1)**	− 6.4 (− 8.5*, − *4.3)**	− 14.1 (− 21.3*, − *6.8)**	− 6.6 (− 9.3*, − *4)**	− 0.6 (− 1.0*, − *0.2) **	− 1.0 (− 1.5*, − *0.5)**	.	− 5.8 (− 9.6*, − *2)**	− 15 (− 22.1*, − *7.8)**
SES				3.8 (0.8*–*6.9)*						
Psychosocial factors										
Household size (> 2 adults)						− 0.2 (− 0.5, − 0)*				.
Family ill member (Yes)				9.6 (5.9–13.4)**	− 2.5 (− 4.1, − 0.8)**					4.5 (0.7–8.2)*
HH factors	Sex of HH head (male)	11.1 (2.3–19.8)*								
	Relation to HH head (child)	− 9.4 (− 17.3*, − *1.5)*						.		
	Age of HH head	11.1 (2.3*–*19.8)*			0.1 (0*–*0.1)*					
CG factors	Sex of CG (male)				.	0.5 (0.1*–*0.9)*				
	Relation of CG to child (mother)				1.7 (0*–*3.4)*					
	Age of CG				.					− 0.2 (− 0.4*, − *0.1)**
	CG child correction (others)				2.6 (0.9*–*4.2)**					.
	CG depression				− 0.03 (− 0.06*–*0.01)		− 0.4 (− 0.7*, − *0.1)*			11.4 (7.7*–*15.2)**
	CG stress			0.3 (0.1*–*0.5)*	0 (− 0.01*–*0) **	− 0.02 (− 0.03*, − *0)**				0.4 (0.1*–*0.6)**
Model diagnostics	Wald chi2	207.2, *P* < 0.001	133.7, *P* < 0.001	88.3, *P* < 0.001	88.8, *P* < 0.001	117.3, *P* < 0.001	190.0, *P* < 0.001	371.7, *P* < 0.001	100.9, *P* < 0.001	103.6, *P* < 0.001
	AIC	4551.9	2971.8	3979.0	3247.1	1477.8	1745.1	2858.9	3497.8	3971.7

All results presented as *aβ* (95% confidence interval)

*SES* socioeconomic status, *HH* household head, *CG* caregiver, *AIC* Akaike's information criteria

*P < 0.05, **P < 0.01…eliminated from the regression model

## Discussion

The primary objective of this study was to evaluate the association between tungiasis and neurocognitive and mental health outcomes in school-aged children. No previous studies have reported the effect of *T. penetrans* infections on neurocognitive and mental health outcomes using validated assessment tools as employed in the current investigation. This study analyses effects of any ectoparasitosis on neurocognitive and behavioural functions in children. This gap of knowledge, which the present study pioneers to close, is particularly problematic since it leads to underestimation of effects parasitic diseases that are highly abundant in poor communities, particularly in the tropics. Similar studies targeting other skin disease such as scabies, pediculosis, and even cutaneous larva migrans caused by hookworms, preferably based on a common set of tools to access neurocognitive and behavioural outcomes, would help recognise the impact of ectoparasitosis on health in children.

A significant association between tungiasis and various neurocognitive domains in school-aged children was observed. Even after adjusting for potential confounders, tungiasis remained significantly associated with poor literacy, language, cognitive flexibility, response inhibition, working memory, fine motor skills, and numeracy scores. In the present study, various other factors were associated with poor neurocognitive outcomes. These included stunting, residing in a specific geographic region, belonging to households of larger size, having a chronically ill family member, relation to the household head, and poor mental health among caregivers. These are well documented risk factors for poor neurocognitive outcomes and for children with tungiasis these factors exacerbate the negative impact of tungiasis on outcomes.

Effects of tungiasis might be either direct (e.g., because pain and itching hamper concentration in school) or indirect (e.g., due to stigmatisation). Potential direct effects due to tungiasis-related pain and itching are obvious but have not been investigated directly. It is well established that pain can affect multiple neurocognitive and behavioural functions [26]. The tungiasis-related stigma has been suggested to negatively impact social interaction and participation in educational activities among affected children. A study in Kenya reported that children with tungiasis experience difficulty in borrowing books from their peers and catching up on missed schoolwork [27]. These findings may partially explain the poor performance in language, attention, memory, perceptual and motor control, and executive functions observed among children with tungiasis in the present study.

We are only beginning to understand the underlying pathophysiology of the impact of tungiasis on neurocognitive ability and mental health in children. However, several hypotheses have been formulated. One possibility is that tungiasis-induced persistent pain may lead to deficits in cognitive flexibility and working memory in children [28]. Furthermore, intense itching and pain caused by tungiasis have been postulated to be linked to poor concentration and sleep disturbances [8]. The relationship between sleep disturbance and cognitive impairment is well-documented [29]. Significant poor performance even after accounting for confounders suggests that other mechanisms, such as upregulation of some host immune responses especially during acute phases and mild stages, are at play. Chronicity and severity of infection, in this case, may desensitize [30] or exhaust [31] these immune mediators, which may partially explain why some neurocognitive scores such as cognitive flexibility and working memory were negatively associated with mild infections and insignificantly associated with severe infections (inferring chronicity). However, none of the published studies directly investigated effects of tungiasis on cognitive and behavioural aspects. The present study found no statistically significant association between tungiasis and mental health outcomes after controlling for other covariates. These results contrast with previous research on other chronic skin disease (atopic dermatitis, psoriasis and vitiligo) which showed a relationship between disease and mental health outcomes [32].

Overall, the findings support the hypothesis that tungiasis has a considerable negative impact on multiple neurocognitive functioning in children and may contribute to neurocognitive impairment. The potential long-term effects of such impairment may include difficulty in learning and performing academic tasks, reduced productivity and earning potential in adulthood, and lower overall quality of life. Of particular interest is the bidirectional relationship between tungiasis neurocognitive and mental health outcomes. The study not only highlights the potential for tungiasis to detrimentally affect cognition and behaviour but also invites consideration of the reverse scenario, where cognitive and behavioural aspects might exert an influence on the occurrence and severity of tungiasis. While this bidirectional perspective adds complexity to the interpretation of the current findings, the study design and analytical approach were primarily geared towards exploring the impact of tungiasis on neurocognitive and mental health outcomes. There is need for further research to understand the mechanisms underlying the observed associations, and to develop effective interventions to mitigate the impact of tungiasis on neurocognitive functioning and overall health in affected children.

One of the limitations of the present study is the fact that presence of other pathogens was not accessed, including parasites such as schistosomes, which are known to be prevalent in the study population and have been previously shown to have cognitive effects [4]. Due to the cross-sectional study design, causality cannot be inferred from the results obtained. Bidirectional causality between tungiasis and neurocognitive function was not thoroughly investigated, with our study primarily focussing on assessing the impact of tungiasis on neurocognitive mental health outcomes. Therefore, future longitudinal research including also potential effects of other infectious diseases is recommended to confirm the effects observed here. Utilizing a combination of neuropsychological and neurophysiological measures would also provide a more comprehensive understanding of the short- and long-term effects of tungiasis on cognitive outcomes in school-aged children. It would also be beneficial to investigate potential mechanisms, such as sleep and attention, through which tungiasis may directly impact cognitive ability.

## Conclusions

This study uncovers the profound detrimental association between tungiasis and the neurocognitive abilities of school-aged children, highlighting a previously overlooked link between this parasitic disease and poor performance across multiple domains. While emphasizing the urgent requirement for comprehensive interventions and targeted support systems, it is important to acknowledge the limitation of not thoroughly investigating the potential bidirectional influence between tungiasis and neurocognitive function. This calls for further research to understand the underlying mechanisms and develop effective interventions aimed at mitigating the impact of tungiasis on children's neurocognitive functioning. The findings strongly advocate for heightened awareness, improved healthcare measures, and resource allocation to effectively address the far-reaching consequences of tungiasis. Failure to address this critical issue perpetuates the cycle of underestimating the devastating effects of parasitic diseases on the cognitive development and overall well-being of vulnerable communities.

### Supplementary Information

**Additional file 1.** Details on neurocognitive domains and behavior problems (S1), tetrachoric PCA procedures (S2), SES measure items (S3), and a scree plot (S4).

**Additional file 2.** The tungiasis dataset containing data collected by Otieno et al for the study of the neurocognitive impact of tungiasis.

## Data Availability

The dataset relevant to the results of this article are provided in the supplementary materials linked to this article as Additional file 2: Otieno et al. Tungiasis Neurocognitive outcome dataset.

## Abbreviations

AIC: Akaike's information criteria
ANCOVA: Analysis of covariance
ANOVA: Analysis of variance
CBCL: Child Behavior Checklist
CDC: Centers for Disease Control and Prevention
CFT: Category fluency test
CG: Caregiver
*CI*: Confidence interval
CTMT: Comprehensive Trail-Making Test
DFG: German Research Foundation
EGMA: Early Grade Maths Assessments
EGRA: Early Grade Reading Assessment
HAZ: Height-for-age Z-scores
HH: Household head
ICIPE: International Centre of Insect Physiology and Ecology
IQR: Interquartile range
KEMRI: Kenya Medical Research Institute
MUAC: Mid-upper arm circumference
PCA: Principal component analysis
PHQ-9: Patient Health Questionnaire-9
PSS: Parent Stress Scale
REDCap: Research Electronic Data Capture
RPM: Raven Progressive Matrices
SCWT: Stroop Color and Word Test
*SD*: Standard deviation
SES: Socioeconomic status
WAZ: Weight-for-age *Z*-scores

## References

[1] Heukelbach J, Oliveira F, Hesse G, Feldmeier H (2001). Tungiasis: a neglected health problem of poor communities. Trop Med Int Health TM IH.

[2] Ugbomoiko US, Ofoezie IE, Heukelbach J (2007). Tungiasis: high prevalence, parasite load, and morbidity in a rural community in Lagos State, Nigeria. Int J Dermatol.

[3] Abubakar A (2014). Biomedical risk, psychosocial influences, and developmental outcomes: lessons from the pediatric HIV population in Africa. New Dir Child Adolesc Dev.

[4] Ezeamama AE, Bustinduy AL, Nkwata AK, Martinez L, Pabalan N, Boivin MJ (2018). Cognitive deficits and educational loss in children with schistosome infection—a systematic review and meta-analysis. PLoS Negl Trop Dis.

[5] Blazevic I (2016). Family, peer and school influence on children’s social development. World J Educ.

[6] Hurt H, Betancourt LM (2016). Effect of socioeconomic status disparity on child language and neural outcome: how early is early?. Pediatr Res.

[7] Feldmeier H, Heukelbach J, Ugbomoiko US, Sentongo E, Mbabazi P, von Samson-Himmelstjerna G (2014). Tungiasis—a neglected disease with many challenges for global public health. PLoS Negl Trop Dis.

[8] Wiese S, Elson L, Feldmeier H (2018). Tungiasis-related life quality impairment in children living in rural Kenya. PLoS Negl Trop Dis.

[9] Okita SY, Seel NM (2012). Social interactions and learning. Encyclopedia of the sciences of learning.

[10] Nampijja M, Apule B, Lule S, Akurut H, Muhangi L, Elliott AM (2010). Adaptation of western measures of cognition for assessing five-year-old semi-urban Ugandan children. Br J Educ Psychol.

[11] StataCorp L. StataCorp stata statistical software: Release 15. StataCorp LP Coll Stn TX USA. 2017.

[12] Gove AK, Wetterberg A (2011). The early grade reading assessment: applications and interventions to improve basic literacy.

[13] Troyer AK (2000). Normative data for clustering and switching on verbal fluency tasks. J Clin Exp Neuropsychol.

[14] Reynolds CR, Spreen O, Strauss E (2002). Comprehensive trail making test: examiner’s manual. A compendium of neuropsychological tests: administration, norms, and commentary.

[15] Scarpina F, Tagini S (2017). The stroop color and word test. Front Psychol.

[16] Jones G, Macken B (2015). Questioning short-term memory and its measurement: why digit span measures long-term associative learning. Cognition.

[17] Schulz J, Henderson S, Sugden D, Barnett A (2011). Structural validity of the Movement ABC-2 test: factor structure comparisons across three age groups. Res Dev Disabil.

[18] Platas LM, Ketterlin-Geller L, Brombacher A, Sitabkhan Y. Early grade mathematics assessment (EGMA) toolkit. RTI Int Res Triangle Park NC. 2014. https://ierc-publicfiles.s3.amazonaws.com/public/resources/EGMA%20Toolkit_March2014.pdf.

[19] John RJ, McCallum RS (2003). Raven progressive matrices. Handbook of nonverbal assessment.

[20] Kitsao-Wekulo PK, Holding PA, Taylor HG, Abubakar A, Connolly K (2013). Neuropsychological testing in a rural African school-age population: evaluating contributions to variability in test performance. Assessment.

[21] Achenbach TM, Kreutzer JS, DeLuca J, Caplan B (2011). Child behavior checklist. Encyclopedia of clinical neuropsychology.

[22] Vidmar S, Carlin J, Hesketh K, Cole T (2004). Standardizing anthropometric measures in children and adolescents with new functions for egen. Stata J.

[23] Kroenke K, Spitzer RL, Williams JB (2001). The PHQ-9: validity of a brief depression severity measure. J Gen Intern Med.

[24] Berry JO, Jones WH (1995). The parental stress scale: initial psychometric evidence. J Soc Pers Relatsh.

[25] Harris PA, Taylor R, Minor BL, Elliott V, Fernandez M, O’Neal L (2019). The REDCap consortium: building an international community of software platform partners. J Biomed Inform.

[26] Whitlock EL, Diaz-Ramirez LG, Glymour MM, Boscardin WJ, Covinsky KE, Smith AK (2017). Association between persistent pain and memory decline and dementia in a longitudinal cohort of elders. JAMA Intern Med.

[27] Ngunjiri JW. Impact of Tungiasis on school age children in Muranga county, Kenya. 2015. http://erepository.uonbi.ac.ke/handle/11295/95003. Accessed 8 Sept 2022.

[28] Beckmann EA, Mano KEJ (2021). Advancing the measurement of executive functioning in pediatric chronic pain. Children.

[29] Mehta KJ (2022). Effect of sleep and mood on academic performance—at interface of physiology, psychology, and education. Humanit Soc Sci Commun.

[30] Schett G, Neurath MF (2018). Resolution of chronic inflammatory disease: universal and tissue-specific concepts. Nat Commun.

[31] Wherry EJ, Kurachi M (2015). Molecular and cellular insights into T cell exhaustion. Nat Rev Immunol.

[32] Dias NG, Caserta Gon MC, Zazula R (2017). Comparison of behavioral profile of children with different chronic skin diseases. Av En Psicol Latinoam.

