# Supplementary material for: Neurocognitive and mental health outcomes in children with tungiasis: a cross-sectional study in rural Kenya and Uganda
Source: Infect Dis Poverty. 2023 Nov 14;12:100. doi: 10.1186/s40249-023-01154-4 (PMC10644620; doi:10.1186/s40249-023-01154-4)
Supplement: Supplementary file 1 — Additional file 1. Details on neurocognitive domains and behavior problems (S1), tetrachoric PCA procedures (S2), SES measure items (S3), and a scree plot (S4). [file 40249_2023_1154_MOESM1_ESM.docx]

**Additional file 1**

**S1 Descriptions of neurocognitive domains and behavior problems**

| **Broad Domains** | **Definition** | **Sub domains** | **Specific Tests used** | **Assessment Procedures** |
| --- | --- | --- | --- | --- |
| **Language** | This domain involves the ability to understand and produce language. Difficulties in this area may manifest as difficulty with comprehension, expressing oneself, or both. (1) | **Literacy ability** | The Early Grade Reading Assessment (EGRA) | Individual participants were presented with printed pages containing letters, words, and sentences and subsequently assessed on key reading competencies such as letter recognition, phonemic awareness, decoding, and comprehension. The assessment scores were calculated based on the total number of correct responses out of a possible 142. |
|  |  | **Language** | Category Fluency Test (CFT) | During the test, participants are asked to generate as many items as possible within a given category, within a limited time frame. In the present study, boys were asked to given names of boys while girls were asked mention names of girls as many as they could within a minute. Scoring was based on the number of correct unique responses. |
| **Attention** | This domain involves the ability to sustain and shift attention, as well as to engage in planning and decision-making. Difficulties in this area may manifest as distractibility, disorganization, or difficulty completing tasks. ^1^ | **Cognitive flexibility** | Comprehensive Trail-Making Test (CTMT) | The test consists of three parts: Trail Making Test A (TMT-A), Trail Making Test B (TMT-B), and Trail Making Test C (TMT-C). In TMT-A, participants were presented with a page with printed numbers and asked to draw lines between consecutive numbers in ascending order as quickly as possible. In TMT-B, participants were presented with a page with printed numbers and letters and asked to connect alternating numbers and letters in ascending order while switching between numbers and letters. TMT-C involved the participant connecting circles with both numbers and letters in an alternating pattern, in ascending order, as quickly as possible while avoiding distractors. The raw scoring of the CTMT is based on the time taken to complete. The raw scores are then reverse transformed adjusting for age of the participant. After transformation, lower scores indicate longer completion times indicating potential cognitive deficits. This study used the transformed mean CTMT scores. |
|  |  | **Response inhibition** | The Stroop Colour and Word Test (SCWT) | In the first trial, participants were presented with a page containing various Colour s printed in black ink and asked to read it (Word Score (W)). Then, in the second trial, participants were shown a page with different Colour patches and were instructed to name the Colour s (Colour Score (C)). Finally, in the third trial, Colour -words were printed in an inconsistent Colour ink, such as the word "red" being printed in green ink. Participants were required to name the Colour of the ink used to print the word instead of reading the word itself (Colour -Word Score (CW)). Each of the trials were timed for 45 seconds. In this study raw interference Score was calculated by subtracting Colour word score from Colour score (CW-C) and transformed based on the age of participants. The transformed interference scores were used to measure attention and response inhibition. |
| **Memory** | This domain involves the ability to acquire and retain new information. Difficulties in this area may manifest as forgetfulness, difficulty with learning new tasks, or difficulty with recall. ^1^ | **Working memory** | The backward digit span task | During the task, the participant is presented with a series of numbers and is asked to repeat them in reverse order. For example, if the sequence presented was "6, 9, 2," the participant would be asked to repeat back "2, 9, 6." The task typically starts with a shorter sequence of numbers and progressively gets longer. In this study, the memory score was the highest level reached by the participant |
| **perceptual-motor control** | This domain involves the ability to perceive, process, and manipulate visual and spatial information. ^1^ Difficulties in this area may manifest as difficulty with spatial orientation, visual recognition, or coordination of movements. ^1^ | **Fine motor ability** | The bead threading test | The participants seated at a table or desk with a bowl of small beads, and a piece of string were asked to thread the beads onto the string as fast as they could. There were three sets each given 30 seconds. The mean number of beads threaded was computed and used as the fine motor control score. |
| **Executive function** | This domain involves the ability to plan, organize, initiate, and monitor complex goal-directed behaviour. ^1^ Difficulties in this area may manifest as difficulty with problem-solving, decision-making, and self-regulation. | **Nonverbal intelligence** | Raven Progressive Matrices (RPM) | The participants were presented with a series of diagrams or patterns that are incomplete and asked to identify the missing piece from a set of options. The scores were based on the number of correct responses. |
|  |  | **Numeracy ability** | Early Grade Maths Ability (EGMA) | The participants were presented with tasks requiring basic mathematical operations and the correct responses totted as numeracy scores. |
| **Behavioural Problems** | All behaviours that concern parents and caregivers | Not applicable | Child Behaviour Checklist (CBCL) | The CBCL consists of a list of statements about the child's behaviour and emotions, which the rater is asked to rate as "Not true", "Somewhat or sometimes true", or "Very true or often true". The statements cover a range of areas including anxiety and depression, aggression and rule-breaking behaviour, attention problems, social skills, and physical complaints. The CBCL provides scores for different domains of behaviour, as well as a total score that reflects overall levels of behavioural and emotional problems. In this study total scores were used to assess behavioural problems, with higher scores indicating more behavioural and emotional problems. |

# S2 Tetrachoric PCA Procedures

| A tetrachoric correlation analysis was conducted in the study to evaluate the relationships among multiple binary variables, including, ownership of radio, TV, cellphone, bicycle1, motorcycle, car, solar lamp, source of drinking water , wall material, toilet type, livestock owned , land owned, roof type, and floor type. Following the analysis, the researchers determined that certain variables (motorcycle, floor, source of water, and car) were strongly correlated with other variables and, thus, excluded from the analysis. The remaining variables ( ownership of radio, TV, cellphone, bicycle, solar lamp, wall material, toilet type, livestock owned , land owned, and roof type,) were then subjected to another tetrachoric correlation analysis. The statistical tests, including rho, se, and p, were utilized, and a Bonferroni correction was applied. The authors also set a significance threshold of 0.05 and ensured that the resulting correlation matrix was positive definite. The variables' internal consistency was assessed using Cronbach's alpha· The suitability of the data for factor analysis was determined using the Bartlett test for sphericity and the Kaiser-Meyer-Olkin test for sampling adequacy· The varimax method was used to rotate the factor loadings to a simpler structure· The first principal component was extracted from the tetrachoric correlation matrix based on eigen value above one and scree plot to create a new variable called the wealth index· |
| --- |

# S3 Items in the SES measure

| **Items** | **Percent of population in each**  **category of ownership (key below)** | | **Factor loadings** |
| --- | --- | --- | --- |
|  | **0** | **1** |  |
| Radio | 33·0 | 67·0 | 0·41 |
| Television | 74·0 | 26·0 | 0·34 |
| Cellphone | 10·6 | 89·4 | 0·34 |
| Bicycle | 52·9 | 47·1 | 0·36 |
| Solar lamp | 38·6 | 61·4 | 0·42 |
| Wall materials ^a^ | 90·1 | 9·9 | 0·1 |
| Toilet facilities ^b^ | 17 | 83·0 | 0·28 |
| Livestock ^c^ | 55·9 | 44·1 | 0·35 |
| Land ownership | 40·5 | 59·5 | 0·18 |
| Roofing materials ^d^ | 35·9 | 64·1 | 0·29 |
| **Diagnostics** | | | |
| Scale reliability coefficient (Cronbach's alpha) | | | 0·62 |
| Bartlett test of sphericity [Chi^2^ *(P-Value*] | | | 398·0, *(<0·001)* |
| Kaiser-Meyer-Olkin Measure of Sampling Adequacy [KMO] | | | 0·73 |

Key: The items are binary with 0=No and 1 =Yes unless specified.^a^ 0 =other materials 1 = Stone, ^b^ 0=bush 1= latrine, ^c^ 0= a few or none 1= several, ^d^ 0=grass/ makuti (palm leaves) thatched 1=other roofing materials

# S4 scree plot


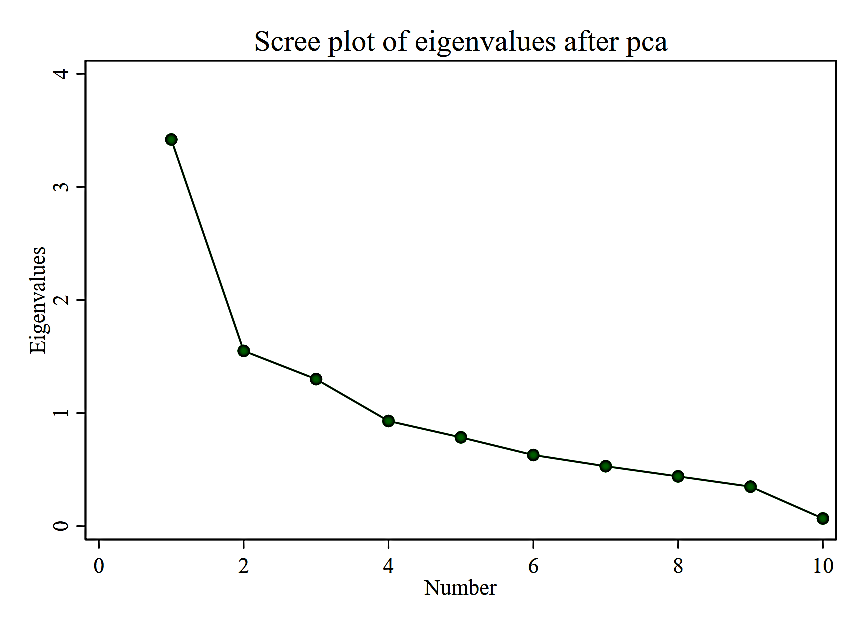


**References**

1. Harvey PD. Domains of cognition and their assessment. *Dialogues Clin Neurosci* [Internet]. 2019 Sep [cited 2023 Jul 13];**21**(3):227–37. Available from: https://www.ncbi.nlm.nih.gov/pmc/articles/PMC6829170/\
